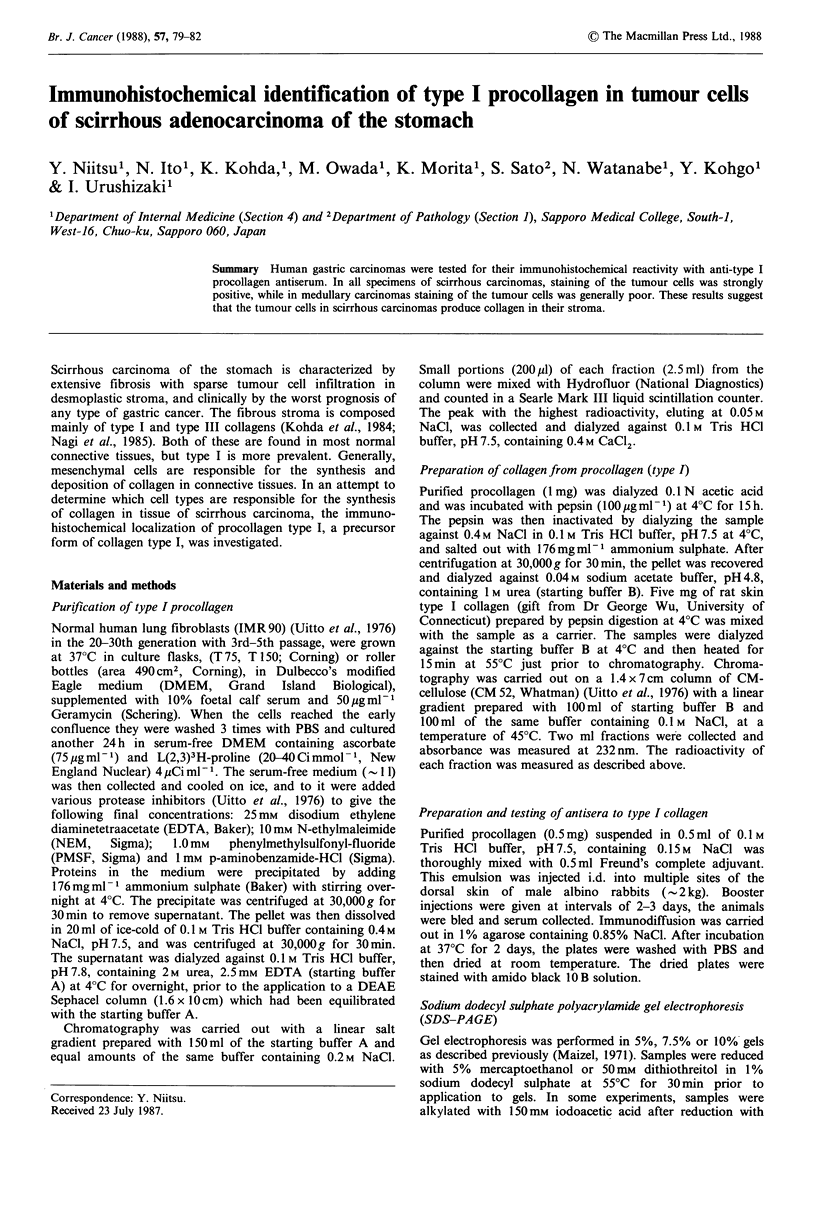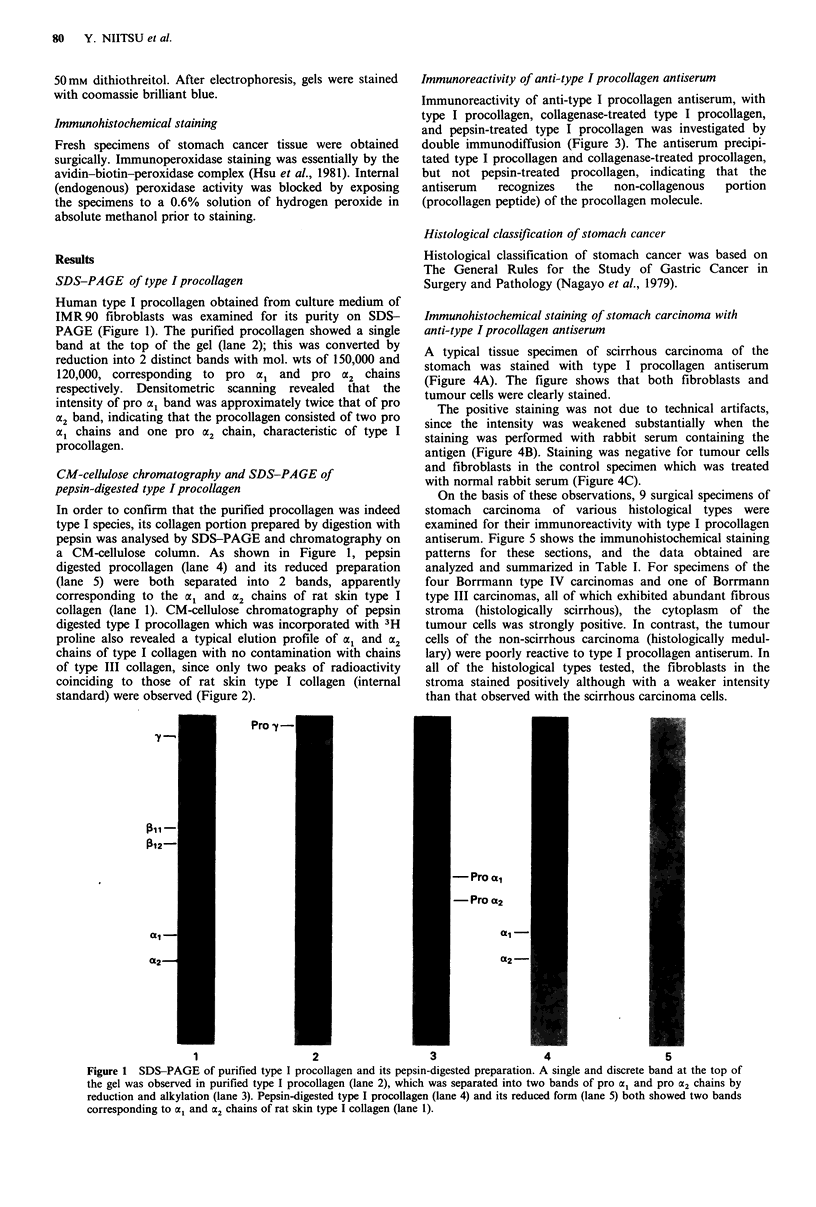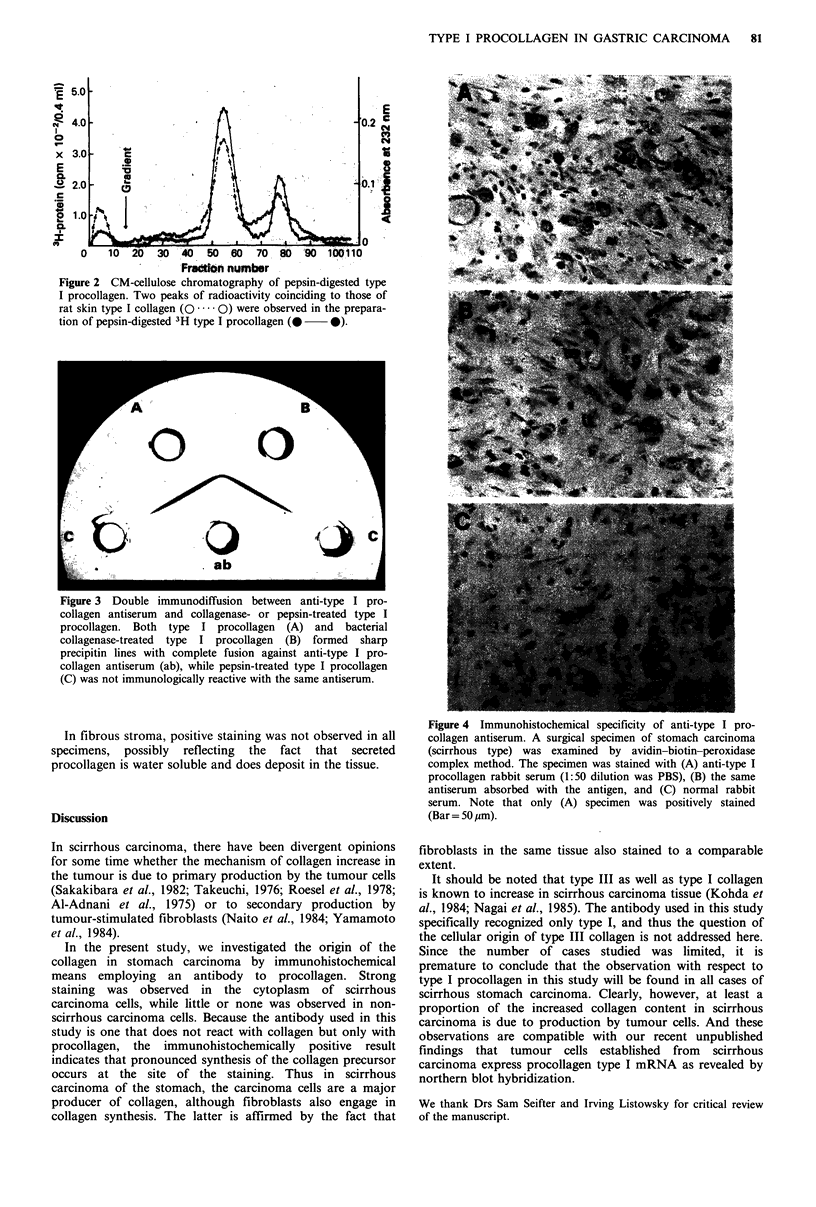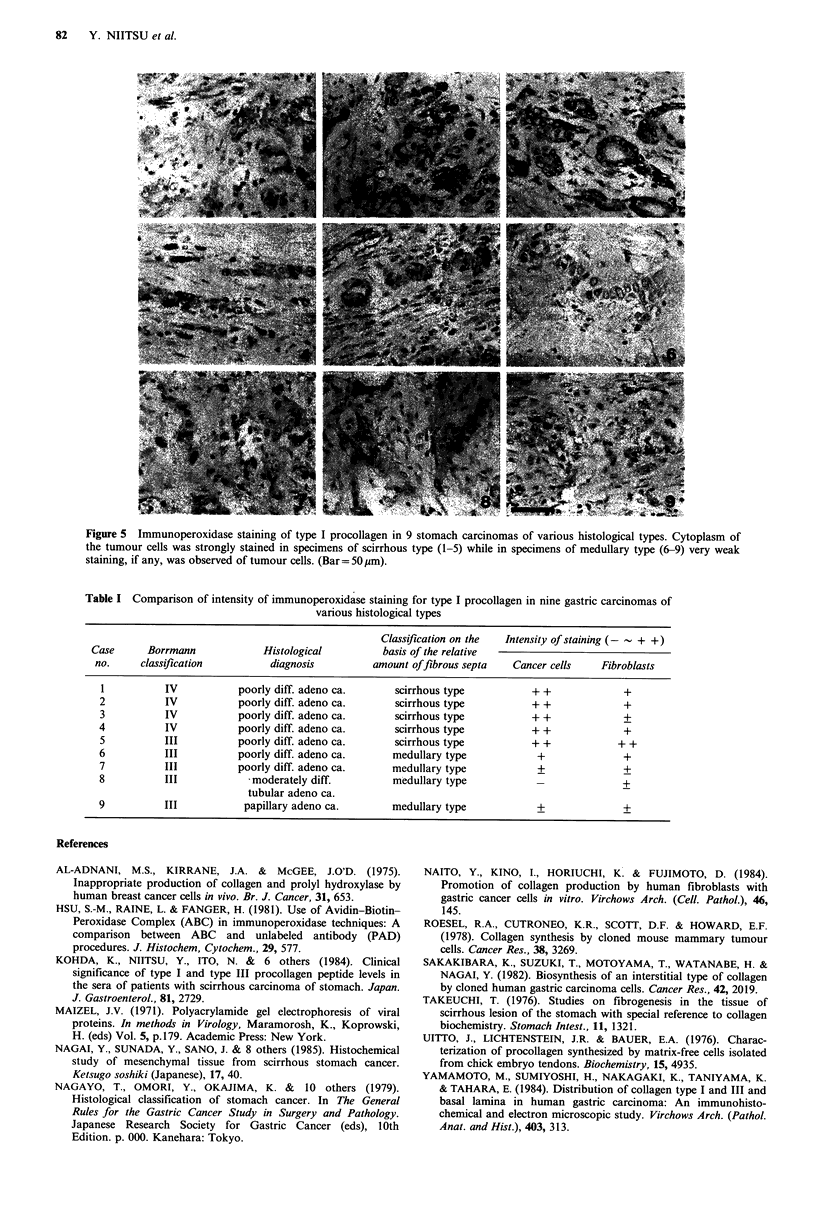# Immunohistochemical identification of type I procollagen in tumour cells of scirrhous adenocarcinoma of the stomach.

**DOI:** 10.1038/bjc.1988.13

**Published:** 1988-01

**Authors:** Y. Niitsu, N. Ito, K. Kohda, M. Owada, K. Morita, S. Sato, N. Watanabe, Y. Kohgo, I. Urushizaki

**Affiliations:** Department of Internal Medicine, Sapporo Medical College, Japan.

## Abstract

**Images:**


*Br. J. Cancer* (1988), **57**, 79–82 © The Macmillan Press Ltd., 1988

### Immunohistochemical identification of type I procollagen in tumour cells of scirrhous adenocarcinoma of the stomach

Y. Niitsu[1], N. Ito[1], K. Kohda,[1], M. Owada[1], K. Morita[1], S. Sato[2], N. Watanabe[1], Y. Kohgo[1] & I. Urushizaki[1]

[1]*Department of Internal Medicine (Section 4) and* [2]*Department of Pathology (Section 1), Sapporo Medical College, South-1, West-16, Chuo-ku, Sapporo 060, Japan*

**Summary** Human gastric carcinomas were tested for their immunohistochemical reactivity with anti-type I procollagen antiserum. In all specimens of scirrhous carcinomas, staining of the tumour cells was strongly positive, while in medullary carcinomas staining of the tumour cells was generally poor. These results suggest that the tumour cells in scirrhous carcinomas produce collagen in their stroma.

Scirrhous carcinoma of the stomach is characterized by extensive fibrosis with sparse tumour cell infiltration in desmoplastic stroma, and clinically by the worst prognosis of any type of gastric cancer. The fibrous stroma is composed mainly of type I and type III collagens (Kohda *et al.*, 1984; Nagi *et al.*, 1985). Both of these are found in most normal connective tissues, but type I is more prevalent. Generally, mesenchymal cells are responsible for the synthesis and deposition of collagen in connective tissues. In an attempt to determine which cell types are responsible for the synthesis of collagen in tissue of scirrhous carcinoma, the immunohistochemical localization of procollagen type I, a precursor form of collagen type I, was investigated.

### Materials and methods

### *Purification of type I procollagen*

Normal human lung fibroblasts (IMR90) (Uitto *et al.*, 1976) in the 20–30th generation with 3rd–5th passage, were grown at 37°C in culture flasks, (T75, T150; Corning) or roller bottles (area 490 $cm^2$, Corning), in Dulbecco's modified Eagle medium (DMEM, Grand Island Biological), supplemented with 10% foetal calf serum and 50 $\mu g\, ml^{-1}$ Geramycin (Schering). When the cells reached the early confluence they were washed 3 times with PBS and cultured another 24 h in serum-free DMEM containing ascorbate (75 $\mu g\, ml^{-1}$) and L(2,3)$^3$H-proline (20–40 Ci $mmol^{-1}$, New England Nuclear) 4 $\mu Ci\, ml^{-1}$. The serum-free medium ($\sim$1 l) was then collected and cooled on ice, and to it were added various protease inhibitors (Uitto *et al.*, 1976) to give the following final concentrations: 25 mM disodium ethylene diaminetetraacetate (EDTA, Baker); 10 mM N-ethylmaleimide (NEM, Sigma); 1.0 mM phenylmethylsulfonyl-fluoride (PMSF, Sigma) and 1 mM p-aminobenzamide-HCl (Sigma). Proteins in the medium were precipitated by adding 176 mg $ml^{-1}$ ammonium sulphate (Baker) with stirring overnight at 4°C. The precipitate was centrifuged at 30,000 *g* for 30 min to remove supernatant. The pellet was then dissolved in 20 ml of ice-cold of 0.1 M Tris HCl buffer containing 0.4 M NaCl, pH 7.5, and was centrifuged at 30,000 *g* for 30 min. The supernatant was dialyzed against 0.1 M Tris HCl buffer, pH 7.8, containing 2 M urea, 2.5 mM EDTA (starting buffer A) at 4°C for overnight, prior to the application to a DEAE Sephacel column (1.6 × 10 cm) which had been equilibrated with the starting buffer A.

Chromatography was carried out with a linear salt gradient prepared with 150 ml of the starting buffer A and equal amounts of the same buffer containing 0.2 M NaCl. Small portions (200 $\mu l$) of each fraction (2.5 ml) from the column were mixed with Hydrofluor (National Diagnostics) and counted in a Searle Mark III liquid scintillation counter. The peak with the highest radioactivity, eluting at 0.05 M NaCl, was collected and dialyzed against 0.1 M Tris HCl buffer, pH 7.5, containing 0.4 M $CaCl_2$.

### *Preparation of collagen from procollagen (type I)*

Purified procollagen (1 mg) was dialyzed 0.1 N acetic acid and was incubated with pepsin (100 $\mu g\, ml^{-1}$) at 4°C for 15 h. The pepsin was then inactivated by dialyzing the sample against 0.4 M NaCl in 0.1 M Tris HCl buffer, pH 7.5 at 4°C, and salted out with 176 mg $ml^{-1}$ ammonium sulphate. After centrifugation at 30,000 *g* for 30 min, the pellet was recovered and dialyzed against 0.04 M sodium acetate buffer, pH 4.8, containing 1 M urea (starting buffer B). Five mg of rat skin type I collagen (gift from Dr George Wu, University of Connecticut) prepared by pepsin digestion at 4°C was mixed with the sample as a carrier. The samples were dialyzed against the starting buffer B at 4°C and then heated for 15 min at 55°C just prior to chromatography. Chromatography was carried out on a 1.4 × 7 cm column of CM-cellulose (CM 52, Whatman) (Uitto *et al.*, 1976) with a linear gradient prepared with 100 ml of starting buffer B and 100 ml of the same buffer containing 0.1 M NaCl, at a temperature of 45°C. Two ml fractions were collected and absorbance was measured at 232 nm. The radioactivity of each fraction was measured as described above.

### *Preparation and testing of antisera to type I collagen*

Purified procollagen (0.5 mg) suspended in 0.5 ml of 0.1 M Tris HCl buffer, pH 7.5, containing 0.15 M NaCl was thoroughly mixed with 0.5 ml Freund's complete adjuvant. This emulsion was injected i.d. into multiple sites of the dorsal skin of male albino rabbits ($\sim$2 kg). Booster injections were given at intervals of 2–3 days, the animals were bled and serum collected. Immunodiffusion was carried out in 1% agarose containing 0.85% NaCl. After incubation at 37°C for 2 days, the plates were washed with PBS and then dried at room temperature. The dried plates were stained with amido black 10 B solution.

### *Sodium dodecyl sulphate polyacrylamide gel electrophoresis (SDS–PAGE)*

Gel electrophoresis was performed in 5%, 7.5% or 10% gels as described previously (Maizel, 1971). Samples were reduced with 5% mercaptoethanol or 50 mM dithiothreitol in 1% sodium dodecyl sulphate at 55°C for 30 min prior to application to gels. In some experiments, samples were alkylated with 150 mM iodoacetic acid after reduction with

Correspondence: Y. Niitsu.
Received 23 July 1987.

50 mM dithiothreitol. After electrophoresis, gels were stained with coomassie brilliant blue.

*Immunohistochemical staining*

Fresh specimens of stomach cancer tissue were obtained surgically. Immunoperoxidase staining was essentially by the avidin–biotin–peroxidase complex (Hsu *et al.*, 1981). Internal (endogenous) peroxidase activity was blocked by exposing the specimens to a 0.6% solution of hydrogen peroxide in absolute methanol prior to staining.

### Results

*SDS–PAGE of type I procollagen*

Human type I procollagen obtained from culture medium of IMR 90 fibroblasts was examined for its purity on SDS–PAGE (Figure 1). The purified procollagen showed a single band at the top of the gel (lane 2); this was converted by reduction into 2 distinct bands with mol. wts of 150,000 and 120,000, corresponding to pro $\alpha_1$ and pro $\alpha_2$ chains respectively. Densitometric scanning revealed that the intensity of pro $\alpha_1$ band was approximately twice that of pro $\alpha_2$ band, indicating that the procollagen consisted of two pro $\alpha_1$ chains and one pro $\alpha_2$ chain, characteristic of type I procollagen.

*CM-cellulose chromatography and SDS–PAGE of pepsin-digested type I procollagen*

In order to confirm that the purified procollagen was indeed type I species, its collagen portion prepared by digestion with pepsin was analysed by SDS–PAGE and chromatography on a CM-cellulose column. As shown in Figure 1, pepsin digested procollagen (lane 4) and its reduced preparation (lane 5) were both separated into 2 bands, apparently corresponding to the $\alpha_1$ and $\alpha_2$ chains of rat skin type I collagen (lane 1). CM-cellulose chromatography of pepsin digested type I procollagen which was incorporated with $^3$H proline also revealed a typical elution profile of $\alpha_1$ and $\alpha_2$ chains of type I collagen with no contamination with chains of type III collagen, since only two peaks of radioactivity coinciding to those of rat skin type I collagen (internal standard) were observed (Figure 2).

*Immunoreactivity of anti-type I procollagen antiserum*

Immunoreactivity of anti-type I procollagen antiserum, with type I procollagen, collagenase-treated type I procollagen, and pepsin-treated type I procollagen was investigated by double immunodiffusion (Figure 3). The antiserum precipitated type I procollagen and collagenase-treated procollagen, but not pepsin-treated procollagen, indicating that the antiserum recognizes the non-collagenous portion (procollagen peptide) of the procollagen molecule.

*Histological classification of stomach cancer*

Histological classification of stomach cancer was based on The General Rules for the Study of Gastric Cancer in Surgery and Pathology (Nagayo *et al.*, 1979).

*Immunohistochemical staining of stomach carcinoma with anti-type I procollagen antiserum*

A typical tissue specimen of scirrhous carcinoma of the stomach was stained with type I procollagen antiserum (Figure 4A). The figure shows that both fibroblasts and tumour cells were clearly stained.

The positive staining was not due to technical artifacts, since the intensity was weakened substantially when the staining was performed with rabbit serum containing the antigen (Figure 4B). Staining was negative for tumour cells and fibroblasts in the control specimen which was treated with normal rabbit serum (Figure 4C).

On the basis of these observations, 9 surgical specimens of stomach carcinoma of various histological types were examined for their immunoreactivity with type I procollagen antiserum. Figure 5 shows the immunohistochemical staining patterns for these sections, and the data obtained are analyzed and summarized in Table I. For specimens of the four Borrmann type IV carcinomas and one of Borrmann type III carcinomas, all of which exhibited abundant fibrous stroma (histologically scirrhous), the cytoplasm of the tumour cells was strongly positive. In contrast, the tumour cells of the non-scirrhous carcinoma (histologically medullary) were poorly reactive to type I procollagen antiserum. In all of the histological types tested, the fibroblasts in the stroma stained positively although with a weaker intensity than that observed with the scirrhous carcinoma cells.

**Figure 1** SDS–PAGE of purified type I procollagen and its pepsin-digested preparation. A single and discrete band at the top of the gel was observed in purified type I procollagen (lane 2), which was separated into two bands of pro $\alpha_1$ and pro $\alpha_2$ chains by reduction and alkylation (lane 3). Pepsin-digested type I procollagen (lane 4) and its reduced form (lane 5) both showed two bands corresponding to $\alpha_1$ and $\alpha_2$ chains of rat skin type I collagen (lane 1).

**Figure 2** CM-cellulose chromatography of pepsin-digested type I procollagen. Two peaks of radioactivity coinciding to those of rat skin type I collagen (○ ···· ○) were observed in the preparation of pepsin-digested $^{3}H$ type I procollagen (● —— ●).

**Figure 3** Double immunodiffusion between anti-type I procollagen antiserum and collagenase- or pepsin-treated type I procollagen. Both type I procollagen (A) and bacterial collagenase-treated type I procollagen (B) formed sharp precipitin lines with complete fusion against anti-type I procollagen antiserum (ab), while pepsin-treated type I procollagen (C) was not immunologically reactive with the same antiserum.

**Figure 4** Immunohistochemical specificity of anti-type I procollagen antiserum. A surgical specimen of stomach carcinoma (scirrhous type) was examined by avidin–biotin–peroxidase complex method. The specimen was stained with (A) anti-type I procollagen rabbit serum (1:50 dilution was PBS), (B) the same antiserum absorbed with the antigen, and (C) normal rabbit serum. Note that only (A) specimen was positively stained (Bar = 50 μm).

In fibrous stroma, positive staining was not observed in all specimens, possibly reflecting the fact that secreted procollagen is water soluble and does deposit in the tissue.

### Discussion

In scirrhous carcinoma, there have been divergent opinions for some time whether the mechanism of collagen increase in the tumour is due to primary production by the tumour cells (Sakakibara *et al.*, 1982; Takeuchi, 1976; Roesel *et al.*, 1978; Al-Adnani *et al.*, 1975) or to secondary production by tumour-stimulated fibroblasts (Naito *et al.*, 1984; Yamamoto *et al.*, 1984).

In the present study, we investigated the origin of the collagen in stomach carcinoma by immunohistochemical means employing an antibody to procollagen. Strong staining was observed in the cytoplasm of scirrhous carcinoma cells, while little or none was observed in non-scirrhous carcinoma cells. Because the antibody used in this study is one that does not react with collagen but only with procollagen, the immunohistochemically positive result indicates that pronounced synthesis of the collagen precursor occurs at the site of the staining. Thus in scirrhous carcinoma of the stomach, the carcinoma cells are a major producer of collagen, although fibroblasts also engage in collagen synthesis. The latter is affirmed by the fact that fibroblasts in the same tissue also stained to a comparable extent.

It should be noted that type III as well as type I collagen is known to increase in scirrhous carcinoma tissue (Kohda *et al.*, 1984; Nagai *et al.*, 1985). The antibody used in this study specifically recognized only type I, and thus the question of the cellular origin of type III collagen is not addressed here. Since the number of cases studied was limited, it is premature to conclude that the observation with respect to type I procollagen in this study will be found in all cases of scirrhous stomach carcinoma. Clearly, however, at least a proportion of the increased collagen content in scirrhous carcinoma is due to production by tumour cells. And these observations are compatible with our recent unpublished findings that tumour cells established from scirrhous carcinoma express procollagen type I mRNA as revealed by northern blot hybridization.

We thank Drs Sam Seifter and Irving Listowsky for critical review of the manuscript.

**Figure 5** Immunoperoxidase staining of type I procollagen in 9 stomach carcinomas of various histological types. Cytoplasm of the tumour cells was strongly stained in specimens of scirrhous type (1–5) while in specimens of medullary type (6–9) very weak staining, if any, was observed of tumour cells. (Bar = 50 μm).

**Table I** Comparison of intensity of immunoperoxidase staining for type I procollagen in nine gastric carcinomas of various histological types

| *Case no.* | *Borrmann classification* | *Histological diagnosis* | *Classification on the basis of the relative amount of fibrous septa* | *Intensity of staining (– ~ + +)* | |
|---|---|---|---|---|---|
| | | | | *Cancer cells* | *Fibroblasts* |
| 1 | IV | poorly diff. adeno ca. | scirrhous type | + + | + |
| 2 | IV | poorly diff. adeno ca. | scirrhous type | + + | + |
| 3 | IV | poorly diff. adeno ca. | scirrhous type | + + | ± |
| 4 | IV | poorly diff. adeno ca. | scirrhous type | + + | + |
| 5 | III | poorly diff. adeno ca. | scirrhous type | + + | + + |
| 6 | III | poorly diff. adeno ca. | medullary type | + | + |
| 7 | III | poorly diff. adeno ca. | medullary type | ± | ± |
| 8 | III | moderately diff. tubular adeno ca. | medullary type | – | ± |
| 9 | III | papillary adeno ca. | medullary type | ± | ± |

### References

AL-ADNANI, M.S., KIRRANE, J.A. & McGEE, J.O'D. (1975). Inappropriate production of collagen and prolyl hydroxylase by human breast cancer cells *in vivo*. *Br. J. Cancer*, **31**, 653.

HSU, S.-M., RAINE, L. & FANGER, H. (1981). Use of Avidin–Biotin–Peroxidase Complex (ABC) in immunoperoxidase techniques: A comparison between ABC and unlabeled antibody (PAD) procedures. *J. Histochem, Cytochem.*, **29**, 577.

KOHDA, K., NIITSU, Y., ITO, N. & 6 others (1984). Clinical significance of type I and type III procollagen peptide levels in the sera of patients with scirrhous carcinoma of stomach. *Japan. J. Gastroenterol.*, **81**, 2729.

MAIZEL, J.V. (1971). Polyacrylamide gel electrophoresis of viral proteins. *In methods in Virology*, Maramorosh, K., Koprowski, H. (eds) Vol. **5**, p.179. Academic Press: New York.

NAGAI, Y., SUNADA, Y., SANO, J. & 8 others (1985). Histochemical study of mesenchymal tissue from scirrhous stomach cancer. *Ketsugo soshiki* (Japanese), **17**, 40.

NAGAYO, T., OMORI, Y., OKAJIMA, K. & 10 others (1979). Histological classification of stomach cancer. In *The General Rules for the Gastric Cancer Study in Surgery and Pathology*. Japanese Research Society for Gastric Cancer (eds), 10th Edition. p. 000. Kanehara: Tokyo.

NAITO, Y., KINO, I., HORIUCHI, K. & FUJIMOTO, D. (1984). Promotion of collagen production by human fibroblasts with gastric cancer cells *in vitro*. *Virchows Arch.* (*Cell. Pathol.*), **46**, 145.

ROESEL, R.A., CUTRONEO, K.R., SCOTT, D.F. & HOWARD, E.F. (1978). Collagen synthesis by cloned mouse mammary tumour cells. *Cancer Res.*, **38**, 3269.

SAKAKIBARA, K., SUZUKI, T., MOTOYAMA, T., WATANABE, H. & NAGAI, Y. (1982). Biosynthesis of an interstitial type of collagen by cloned human gastric carcinoma cells. *Cancer Res.*, **42**, 2019.

TAKEUCHI, T. (1976). Studies on fibrogenesis in the tissue of scirrhous lesion of the stomach with special reference to collagen biochemistry. *Stomach Intest.*, **11**, 1321.

UITTO, J., LICHTENSTEIN, J.R. & BAUER, E.A. (1976). Characterization of procollagen synthesized by matrix-free cells isolated from chick embryo tendons. *Biochemistry*, **15**, 4935.

YAMAMOTO, M., SUMIYOSHI, H., NAKAGAKI, K., TANIYAMA, K. & TAHARA, E. (1984). Distribution of collagen type I and III and basal lamina in human gastric carcinoma: An immunohistochemical and electron microscopic study. *Virchows Arch.* (*Pathol. Anat. and Hist.*), **403**, 313.